# A Rare Case of Waterhouse-Friderichsen Syndrome Without Purpura Secondary to Haemophilus Influenzae

**DOI:** 10.7759/cureus.9621

**Published:** 2020-08-09

**Authors:** Lawman Chiwome

**Affiliations:** 1 General Internal Medicine, University Hospitals of Morecambe Bay NHS Foundation Trust, Lancaster, GBR

**Keywords:** adrenal insufficiency, bilateral adrenal haemorrhage, waterhouse-friderichsen syndrome, haemophilus influenzae

## Abstract

The Waterhouse-Friderichsen syndrome is an entity consisting of shock, petechial rash and haemorrhages in both adrenal glands leading to adrenal failure. This syndrome is usually secondary to meningococcal septicaemia, but there are many documented cases caused by other bacteria. Purpura is an essential part of the syndrome, but it is not always there. In the current study, a case of Waterhouse-Friderichsen syndrome without purpura in an elderly patient with Haemophilus influenzae bacteraemia has been described. This patient was being managed for sepsis due to pneumonia and an incidental finding of bilateral adrenal haemorrhage was made on a CT of the thorax which was meant to evaluate empyema. This case shows the need to suspect bilateral adrenal haemorrhage in every patient with septic shock.

## Introduction

The Waterhouse-Friderichsen syndrome (WFS) is an entity consisting of shock, petechial rash and haemorrhages in both adrenal glands leading to adrenal failure [1-3]. This syndrome is usually secondary to meningococcal septicaemia, but there are many documented cases caused by other bacteria. It is mostly diagnosed at post-mortem. Waterhouse-Friderichsen syndrome, named after Rupert Waterhouse, an English Physician and Carl Friderichsen, after they reviewed cases in 1911 and 1918, respectively [4,5]. Purpura is an essential part of the syndrome, but it is not always there [1-3]. Here is a described case of Waterhouse-Friderichsen syndrome without purpura in an elderly patient with Haemophilus influenzae bacteraemia from sepsis due to pneumonia. The bilateral adrenal haemorrhage was an incidental finding on a CT of the thorax. 

## Case presentation

A 72-year-old woman presented with pleuritic chest pain and shortness of breath. She has a background history of breast cancer (treated with mastectomy and chemotherapy) and atrial fibrillation. She was taking apixaban and bisoprolol. On assessment, her blood pressure was 80/60 mmHg, oxygen saturation level was 98% on room air, with a tympanic temperature of 38 degrees Celsius. Chest examination revealed signs consistent with right lower lobe consolidation. All the other systems were essentially normal.

**Figure 1:**
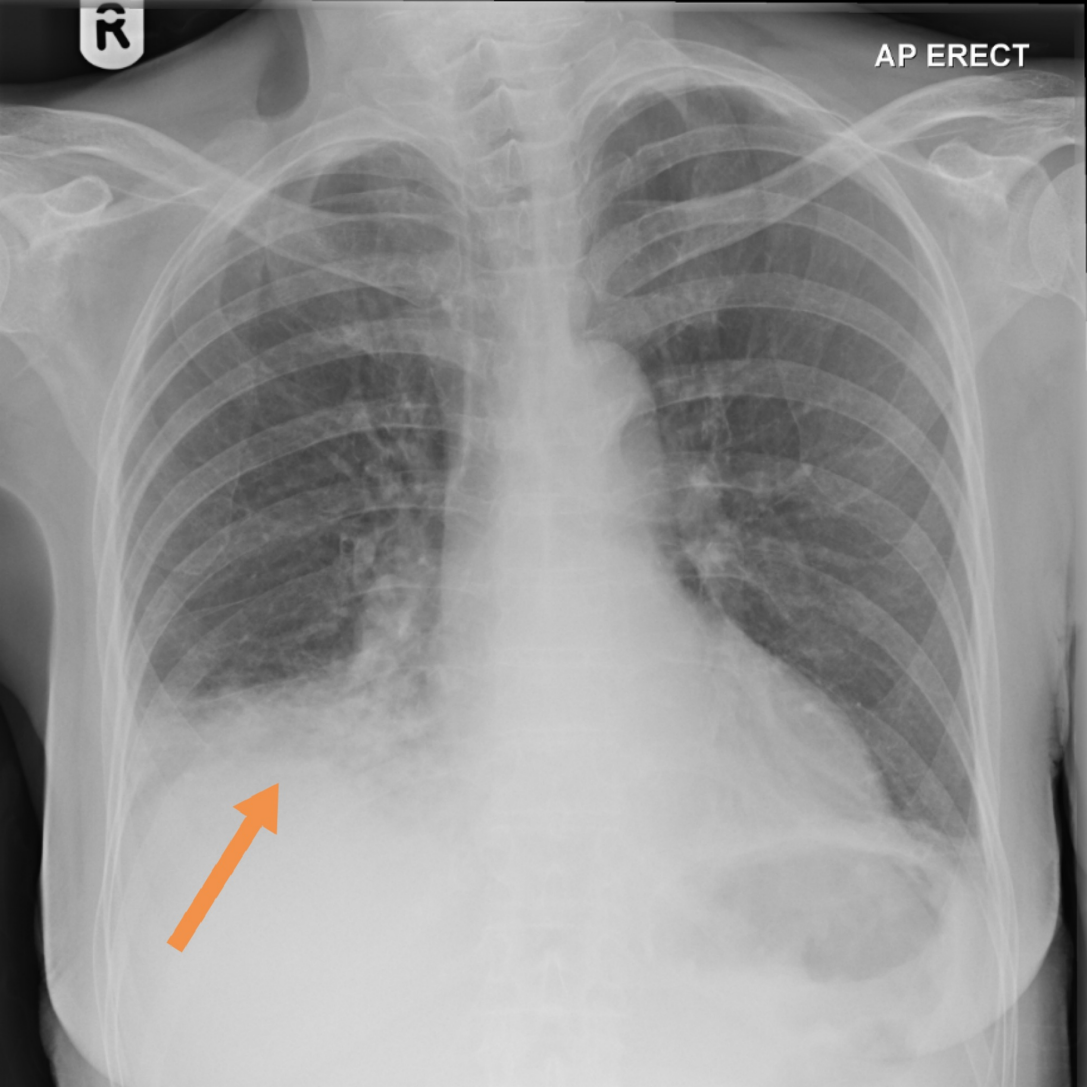
Chest x-ray on admission showing right lower lobe consolidation (see arrow)

Chest x-ray showed right lower lobe consolidation. Blood results showed a high C-reactive protein (CRP) and leucocytosis. Renal function was normal except for low serum sodium of 128 mmol/L. The international normalised ratio (INR) was 3. Sepsis and the fact that she was on apixaban explained the raised INR. Other essential tests done on admission are blood culture and urine culture. Her electrocardiogram (ECG) demonstrated atrial fibrillation. The patient was managed for right lower lobe pneumonia and intravenous co-amoxiclav (1,200 mg three times a day) was initiated. The chest x-ray on admission is shown in Figure 1.

**Figure 2:**
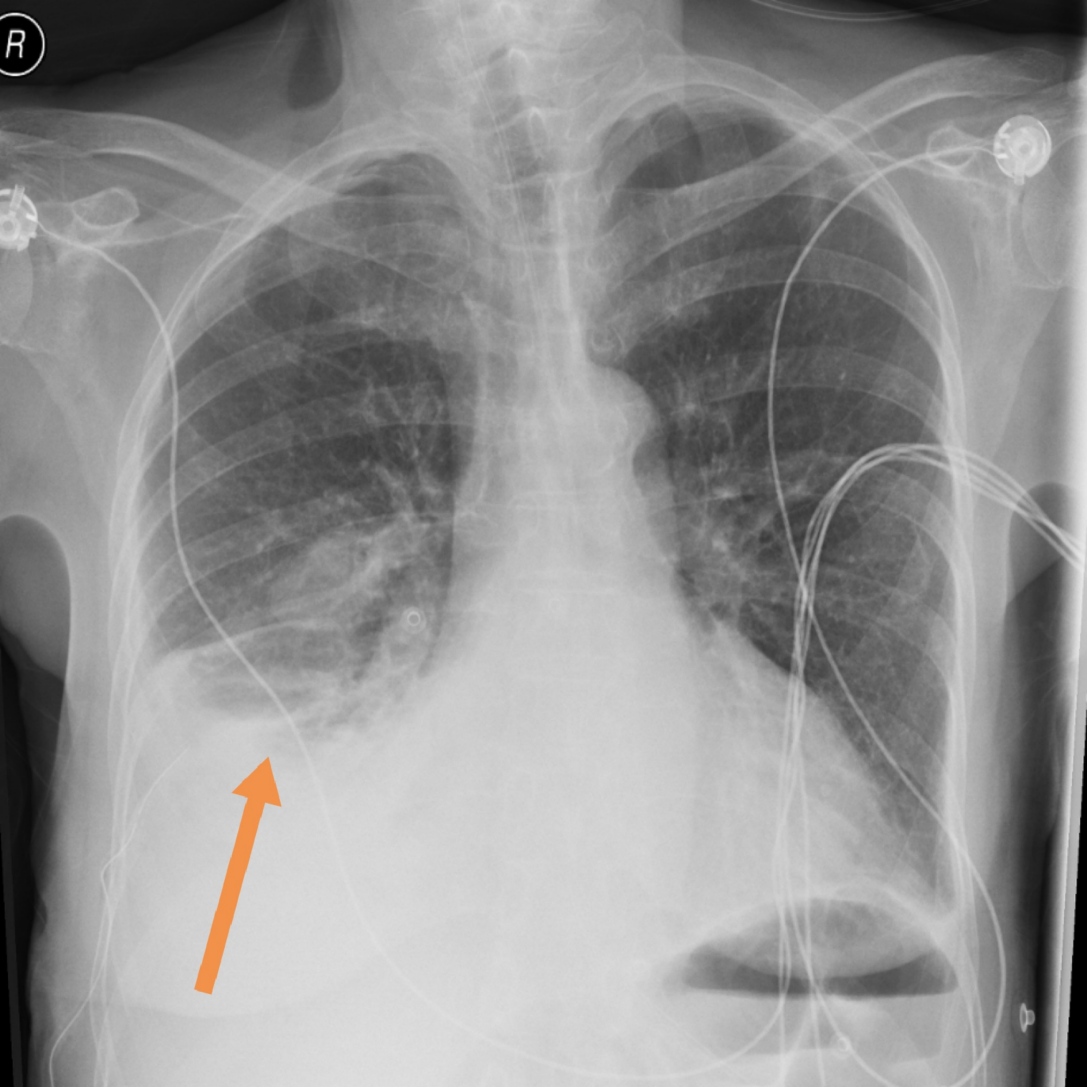
Chest x-ray showing empyema (see arrow)

Throughout her admission, she remained hypotensive but the patient was asymptomatic. Blood culture grew Haemophilus influenzae, and urine culture grew Pseudomonas aeruginosa. The latest culture results led to antibiotics change to ciprofloxacin to cover for both organisms after advice from microbiologist. On day 7, CRP had increased to 250 from 100 mg/L, and white blood cell count (WBC) rose to 9.3 × 10^9^ cells/L from 8.3 × 10^9^ cells/L. Following discussion with a microbiologist, her antibiotic was changed to intravenous ceftriaxone. The repeated chest x-ray showed right-sided pleural effusion, and this led to the insertion of a chest drain. The pleural aspirate demonstrated empyema. The repeat chest x-ray is shown in Figure 2.

**Figure 3:**
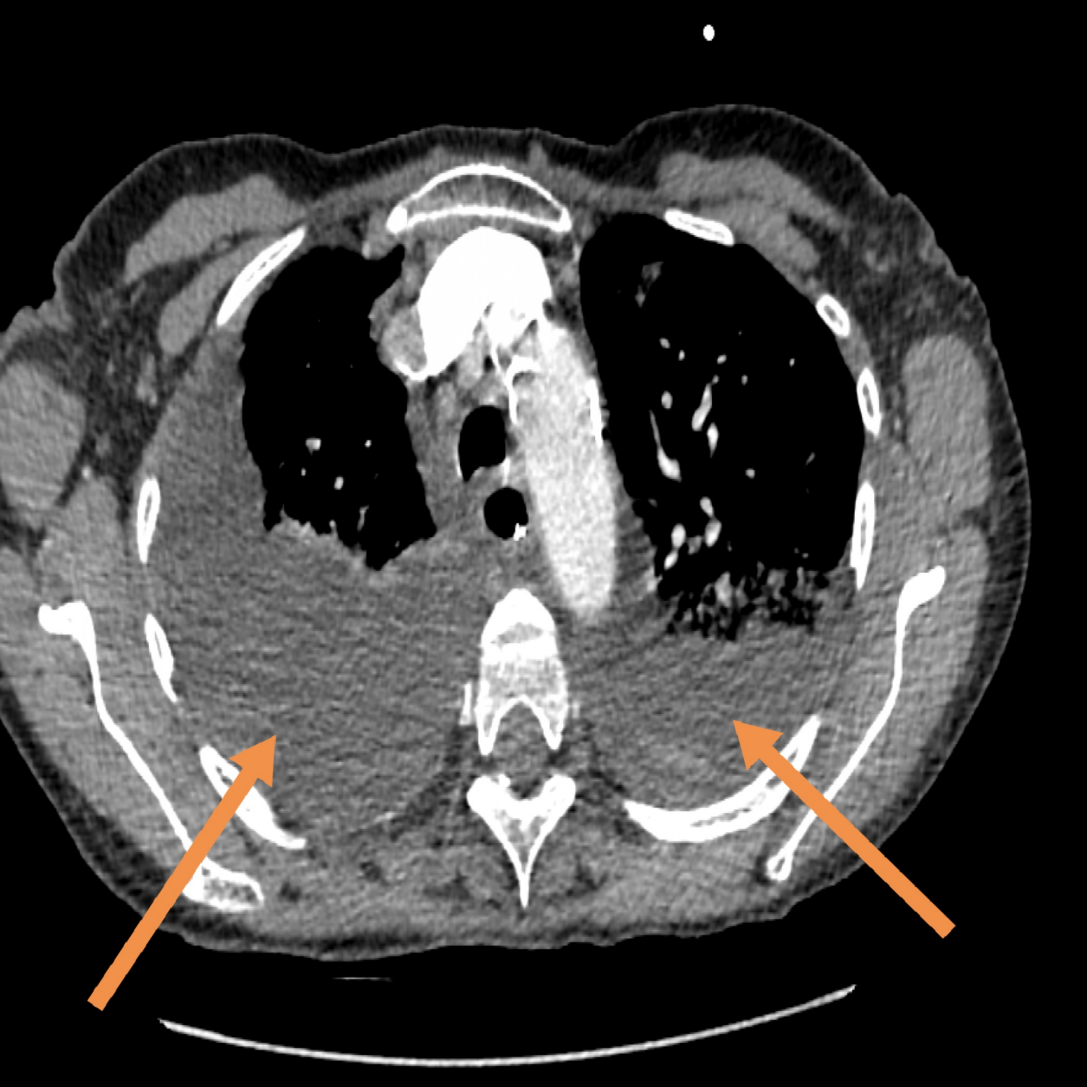
CT of the thorax showing bilateral empyema (see arrows)

**Figure 4:**
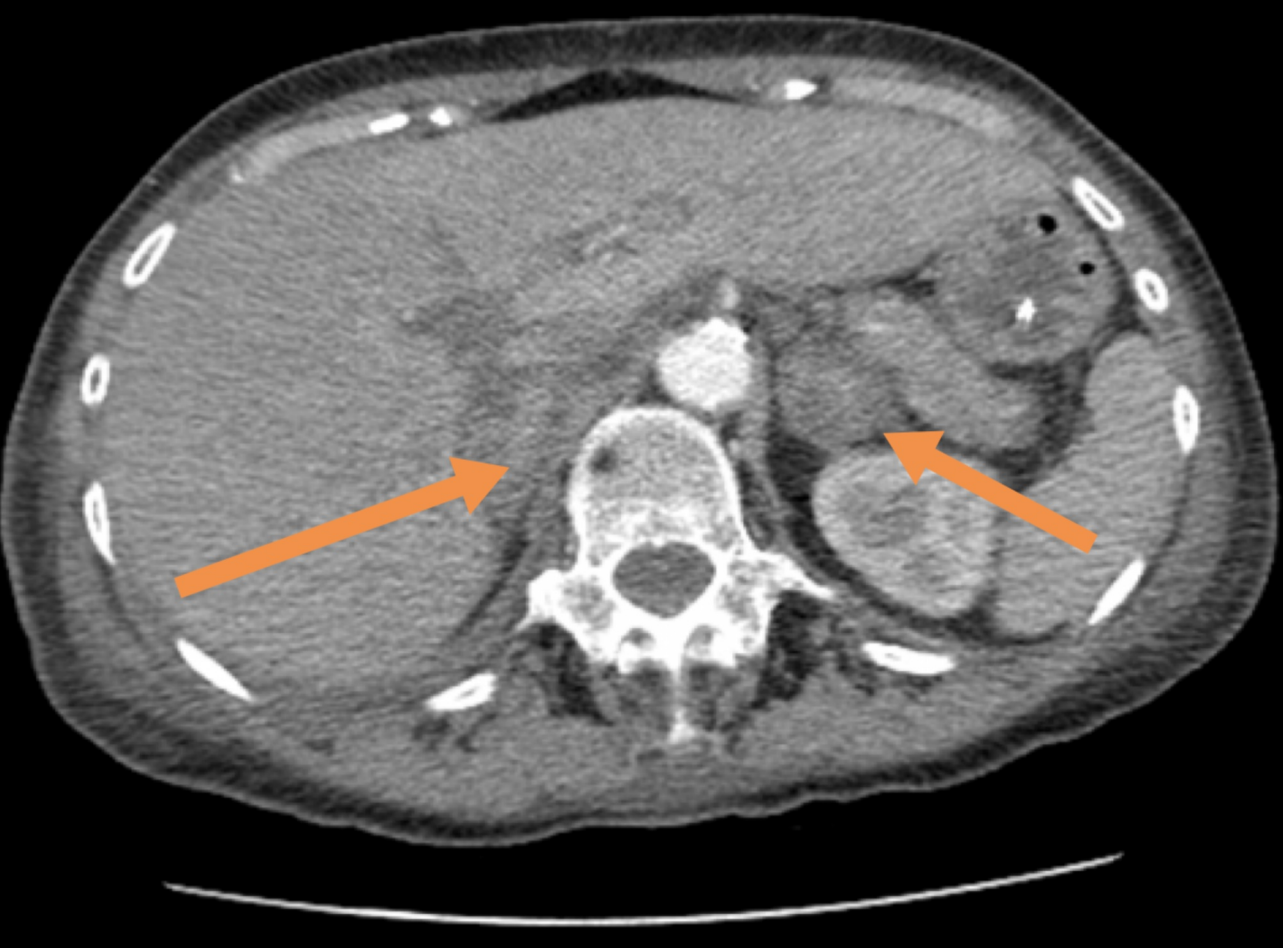
Image showing bilateral adrenal haemorrhage (see arrows)

**Figure 5:**
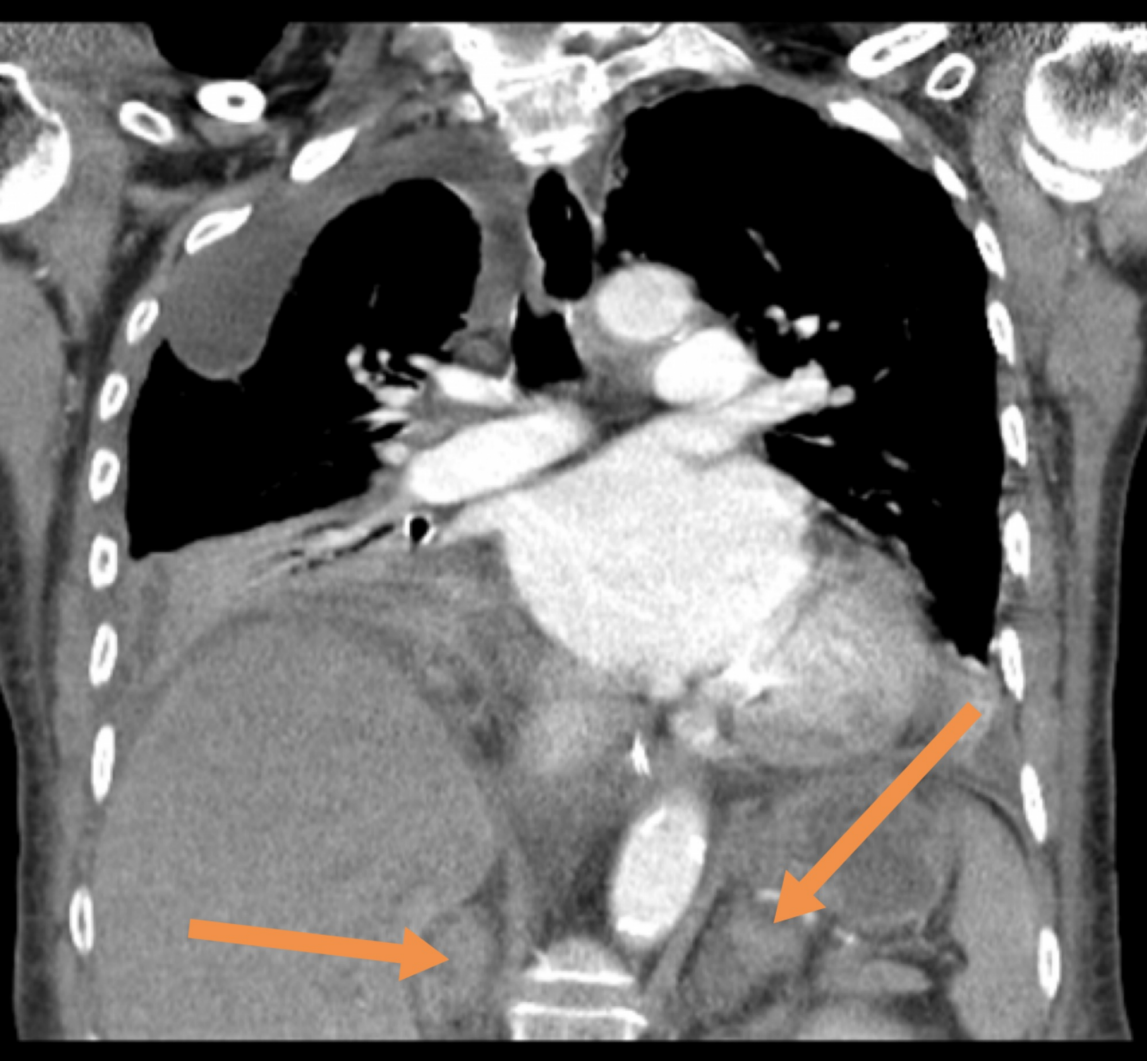
Image showing bilateral adrenal haemorrhage (see arrows)

On day 12, her blood pressure reduced to 75/45 mmHg, which was not responding to fluid challenge so the patient was admitted to intensive therapy unit. Her serum sodium level was 118 mmol/L, urine osmolality 463 mOsm/kg, urine sodium 13 mmol/L, and plasma osmolality 253 mOsm/kg. Tazocin and levofloxacin were initiated. On day 13 post-admission, CT thorax demonstrated worsening bilateral empyema (Figure 3), and there was an incidental finding of bilateral adrenal haemorrhage (Figures 4, 5).

A chest drain was re-inserted to drain the empyema, and the patient was intubated. Cortisol levels were done, and the result was 57 nmol/L suggestive of adrenal insufficiency caused by bilateral adrenal haemorrhage due to severe sepsis. She was started on hydrocortisone 100 mg twice daily. The next day, the patient improved and ventilatory support stopped. Three days later, hydrocortisone was reduced to 50 mg three times a day. She left intensive therapy unit after 10 days in the unit and discharged home after spending 27 days in the hospital. She was discharged on oral hydrocortisone. Six weeks post-discharge, she came back for review, and she was doing well with no concerns raised.

Trend of blood results is shown in Table 1.

**Table 1:** Trend of blood results from day of admission till discharge. HB: haemoglobin; MCV: mean corpuscular volume; PLT: platelet; WBC: white blood cell; K: serum potassium; Na: serum sodium; CRP: C-reactive protein; Ca: serum calcium; Phos: serum phosphate; Mg: serum magnesium; INR: international normalised ratio; g/L: grams per litre; fl: femtolitre; L: litre; mmol/L: millimoles per litre; mg/L: milligrams per litre; ITU: intensive therapy unit

	Normal range	Day 2	Day 4	Day 6	Day 7	Day 11	Day 12 day 1 ITU	Day 13 Day 2 ITU	Day 14	Day 16	Day 17	Day 19	Day 20	Day 21
HB	115-165 g/L	123	121	123	129	127	125	112	107	83	88	97	85	95
MCV	80-100 fl	97	98	95	98	94	94	94	95	94	95	95	93	93
PLT	150-450 × 10^9^/L	188	249	315	359	477	498	459	474	410	409	474	411	481
WBC	04-11 × 10^9^/L	17	8.4	8.3	9.3	11	11	17.9	15	10.7	8	12.4	12.7	12.6
K	3.5-5.1 mmol/L	4.5	4.8	4.4		3.3	4	4.5	4.6	4	4.2	5.4	4.6	3.4
Na	135-145 mmol/L	134	133	132		122	118	121	126	132	133	134	134	135
CRP	<5 mg/L	258	186	250	250	143	161	245	308	246	290	254	88.4	39.3
Ca	2.2-2.6 mmol/L					2.51	2.06	2.36	2.3	1.98	2.43	2.45	2.4	2.39
Phos	0.8-1.5 mmol/L					0.51	0.71	0.81	1.36	1.01	1.07	1.76	0.86	0.6
Mg	0.8-1.0 mmol/L					0.62	0.67	0.66	0.59	0.88	0.72	0.75	0.69	0.69
INR	0.8-1.2	3.1						1.9	2	1.4	1.3	1.4		

## Discussion

Epidemiology and aetiology

The WFS is a rare disease and mostly fatal, and it occurs acutely in most cases [3-9]. It is mainly diagnosed at post-mortem; thus, the need for a high index of suspicion and urgent intervention is required. Neisseria meningitidis is isolated in more than 80% of WFS cases, other bacteria are Haemophilus influenzae and Pseudomonas aeruginosa, as seen in this case, Streptococcus pneumoniae, Staphylococcus aureus, group A Streptococcus, Neisseria gonorrhoeae, Proteus mirabilis, Klebsiella, and Legionella [4-10]. It is essential to know that bilateral adrenal haemorrhage on its own has other causes besides sepsis. These are heart failure, immense physical stress such as trauma and surgery, antiphospholipid syndrome, oral anticoagulants, heparin-induced thrombocytopaenia, underlying adrenal tumour, and pregnancy [7-11]. Sepsis explained the bilateral haemorrhage in this case. It could have been worsened by direct oral anticoagulation as the patient was on apixaban and INR on admission was 3. The cause is unknown sometimes, as evidenced by documented cases of idiopathic bilateral adrenal haemorrhage [5-11].

Bilateral adrenal haemorrhage presents with vague symptoms, and this led to late diagnosis in this case [4-11]. It was diagnosed 10 days after admission as an incidental finding on CT thorax. This shows that no one was suspecting adrenal insufficiency in this case. Late diagnosis is because this patient had septic shock due to pneumonia; severe sepsis and inadequate fluid intake explained the low blood pressure. The diagnosis is so challenging that it is often diagnosed at post-mortem [7-12]. Symptoms of adrenal haemorrhage include abdominal, back, and flank pain. Fever and hypotension are also part of the spectrum. Adrenal haemorrhage can be unilateral or bilateral. One-sided adrenal bleeding is usually asymptomatic, and bilateral haemorrhage has a poor prognosis. It is vital to take a good history and do a thorough examination physical to find historical risk factors for adrenal haemorrhage and have a high index of suspicion in anyone with sepsis [4-12].

Pathophysiology

Sepsis is a serious condition that results in organ dysfunction caused by an unbalanced body response to fighting infection [4-14]. In this case, organ dysfunction is type 1 respiratory failure and circulatory collapse. Sepsis has a high hospital death rate, especially in the elderly and those with comorbidities.

In sepsis, there is an activation of the coagulation system and fibrinolytic pathways and suppression of anticoagulant pathways resulting in thrombosis [4-14]. Infection that causes adrenal haemorrhage is mainly seen with Neisseria meningitides septicaemia, and the term Waterhouse-Friderichsen syndrome is mostly coined for this. This patient presented 10 days before the diagnosis of adrenal haemorrhage with infection caused by Haemophilus influenzae. Lipopolysaccharides on the cell wall of gram-negative bacteria activate the immune system, leading to the release of inflammatory cytokines and other mediators. Despite treatment with antibiotics and steroids, the outcome is still abysmal [4,5,7-14]. Disseminated intravascular coagulation (DIC) results in bleeding in the skin and adrenal glands which then lead to shock [1-14]. The presence of thrombi in organs supports the theory that DIC happens. Endotoxaemia also contributes to the adrenal haemorrhage. After putting all this information together, it is important to consider heparin in the management of this disease. In this case, the patient was on direct oral anticoagulant and was later started on low molecular weight heparin, which could have contributed to the patient's survival. Hyponatraemia in adrenal insufficiency is mainly due to secretion of antidiuretic hormone (ADH). ADH causes water absorption by the kidneys leading to dilutional hyponatraemia. ADH is secreted in response to low blood pressure and low cardiac output and also cortisol is a physiologic inhibitor of ADH [1-8].

Investigations

Classical electrolyte derangements in acute adrenal insufficiency include hyperkalaemia, hyponatraemia, hypocalcaemia, and hypermagnesaemia [3-14]. In our patient, there was hyponatraemia only. The serum potassium and serum magnesium were low, while serum calcium was high at some point. These derangements are due to severe sepsis. Elevated calcium levels led to multiple myeloma investigation of which it was negative. Urine osmolality of 463 mOsm/kg, blood osmolality of 253 mOsm/kg, and urine sodium of 13 mmol/L were consistent with adrenal insufficiency except for the low urine sodium. In classical adrenal insufficiency, serum osmolality is less than 280 mOsm/kg, urine osmolality is greater than 100 mOsm/kg, and urine sodium is greater than 20 mmol/L [1-14].

Cortisol levels were 57 nmol/L, and this was low compared to the stress the patient was in due to the sepsis. There is a significant role for imaging investigations, especially the CT that diagnosed the bilateral adrenal haemorrhage in this case. If there is suspicion for bilateral adrenal haemorrhage, a CT of the abdomen must be done as soon as possible [3-14]. Common findings on CT are masses of variant sizes to clear haemorrhages distorting adrenal architecture.

MRI has a higher sensitivity to pick adrenal haemorrhages and is better than CT because it can differentiate a haematoma from dead tissue. It can also tell us the age of the haematoma [3-14]. However, a CT scan is preferred as an initial investigation of choice [1-14]. In this case, MRI of the adrenal glands was not done.

Management and follow-up

The preferred treatment for WFS is intravenous antibiotics, intravenous hydrocortisone, and fluid resuscitation with normal saline. Despite treatment, the mortality remains very high; this is worsened by delayed diagnosis and initiation of treatment [3-14]. In this case, the patient was started on 100 mg hydrocortisone twice daily, and there was a dramatic improvement. The patient was discharged on oral hydrocortisone and advised on what to expect from impending adrenal crisis.

## Conclusions

This was an incidental finding of bilateral adrenal haemorrhage; therefore, clinicians should consider potential adrenal insufficiency in every patient with septic shock. This was a rare presentation of WFS due to Haemophilus influenzae infection. To diagnose bilateral adrenal haemorrhage, one must have a high index of suspicion, and there is a need for an urgent intervention with corticosteroid therapy and management of septic shock. Finally petechial rash may not be a feature of the syndrome when caused by Haemophilus influenzae. 
